# Military trainees can accurately measure optic nerve sheath diameter after a brief training session

**DOI:** 10.1186/s40779-018-0189-y

**Published:** 2018-12-20

**Authors:** Joseph Betcher, Torben K. Becker, Peter Stoyanoff, Jim Cranford, Nik Theyyunni

**Affiliations:** 1 Department of Emergency Medicine, Mercy Health Muskegon, 1500 E Sherman Blvd, Muskegon, MI 49444 USA; 2 Department of Critical Care Medicine, University of Pittsburgh Medical Center, 200 Lothrop St, Pittsburgh, PA 15213 USA; 3 Department of Emergency Medicine, Hurley Hospital, 1 Hurley Plaza, Flint, MI 48503 USA; 4 Department of Psychiatry, University of Michigan, 1500 E Medical Center, Ann Arbor, MI 48109 USA; 5 Department of Emergency Medicine, University of Michigan, 1500 E Medical Center, Ann Arbor, MI 48109 USA

**Keywords:** Ultrasound, Military, Optic nerve sheath diameter, Intracranial pressure, Education

## Abstract

**Background:**

Identification of elevated intracranial pressure is important following traumatic brain injury. We assessed the feasibility of educating military trainees on accurately obtaining optic nerve sheath diameter measurements using a brief didactic and hands-on training session. Optic nerve sheath diameter is a noninvasive surrogate marker for elevated intracranial pressure, and may be of value in remote military operations, where rapid triage decisions must be made without access to advanced medical equipment.

**Methods:**

Military trainees with minimal ultrasound experience were given a 5-min didactic presentation on optic nerve sheath diameter ultrasound. Trainees practiced optic nerve sheath diameter measurements guided by emergency physician ultrasound experts. Trainees then measured the optic nerve sheath diameter on normal volunteers. Following this, a trained physician measured the optic nerve sheath diameter on the same volunteer as a criterion standard. An average of three measurements was taken.

**Results:**

Twenty-three military trainees were enrolled. A mixed design ANOVA was used to compare measurements by trainees to those of physicians, with a mean difference of − 0.6 mm (*P* = 0.76). A Bland-Altman analysis showed that the degree of bias in optic nerve sheath diameter measures provided by trainees was very small: *d* = − 0.004 for the right eye and *d* = − 0.007 for the left eye.

**Conclusion:**

This study demonstrates that optic nerve sheath diameter measurement can be accurately performed by novice ultrasonographers after a brief training session. If validated, point-of-care optic nerve sheath diameter measurement could impact the triage of injured patients in remote areas.

## Background

Elevated intracranial pressure (ICP) is a condition that leads to increased morbidity and mortality. Closed head injury following blunt trauma is one of the most common indications for ICP monitoring. Elevated ICP leads to decreased cerebral blood flow, and poor neurologic outcomes in patients. Early detection of elevated ICP can guide therapy and management, and therefore elevated ICP is especially important and can be problematic in the military and in the field in particular.

Invasive monitoring is the gold standard but has multiple disadvantages, including infection, which occurs in ~ 10% of patients [1, 2]. Risk of infection increases the longer a device is left in a patient. Additionally, 2% of patients develop intraventricular hemorrhage during placement and removal of the device [3]. Specialists, such as neurosurgeons, are required to place these devices. In resource-limited areas, specialists with advanced training and equipment may not be available. Ultrasound to measure optic nerve sheath diameter (ONSD) has been proven to accurately and noninvasively screen for elevated ICP in previous studies [4, 5]. There is also a suggestion that ONSD measurements can be used in evaluation of brain death [6].

Ultrasound confers no risk of infection or bleeding and can be performed in minutes. ONSD requires only minimal skill, basic ultrasound knowledge and is taught without difficulty [7]. This measurement has been previously proven to correlate with invasive ICP monitoring [8]. This modality has also been previously used reliably in intensive care units for ICP monitoring when more advanced monitoring was not available [9]. Teaching this skill to combat medics may prevent the need for invasive, expensive, and time consuming procedures such as invasive intracranial monitoring and, more importantly, may help guide rapid triage, treatment and evacuation decisions in remote or combat environments where access to specialists and advanced medical equipment is limited or nonexistent.

Previous studies have shown good intra and interobserver reliability with measurements of the ONSD [10, 11]. However, other studies have shown some variation in agreements between ONSD measurements among physicians, while still demonstrating good interrater reliability [12]. The precise number of scans needed to become proficient in this ultrasound application has been suggested to range from 10 scans in those proficient with point-of-care ultrasound to 25 scans for novices [13]. Additional work has suggested that scanning approximately 20 subjects for ONSD is the number at which skill level appears to plateau, although this was measured in only a single provider [14].

We attempted to evaluate military trainees’ ability to measure the ONSD in healthy volunteers after attending a very brief training session, to evaluate whether or not a novice sonographer can accurately perform ONSD.

## Methods

The study was open to Special Operations Combat Medic (SOCM) trainees who performed monthly training rotations within the emergency department during their SOCM training. Participation was voluntary, and no compensation was offered. We conducted this proof-of-concept study within a classroom setting in the emergency department. The study was reviewed by the institutional review board under study number 601973–2 and was found to be exempt from full and ongoing review. Informed consent was obtained from all participants and was approved by the institutional review board. Mindray® M7 machines (Mindray Ltd., Shenzhen, CN.) available to the emergency department for regular patient care use were utilized. Instructors were emergency physician ultrasound faculty or senior emergency physician residents with extensive additional training in ultrasound. A 5-min lecture introduced the concept and technique of ONSD measurements to the SOCM trainees, and then demonstrated the technique in small groups. SOCM trainees practiced the technique in the axial view on other healthy SOCM trainees, with hands-on instruction from the instructors for 20 min. The linear probe, along with the superficial setting, was utilized. Trainees practiced both performing scans on other SOCM trainees and taking measurements of the ONSD with hands-on instruction from the instructors for a total of 20 min.

**Fig. 1:**
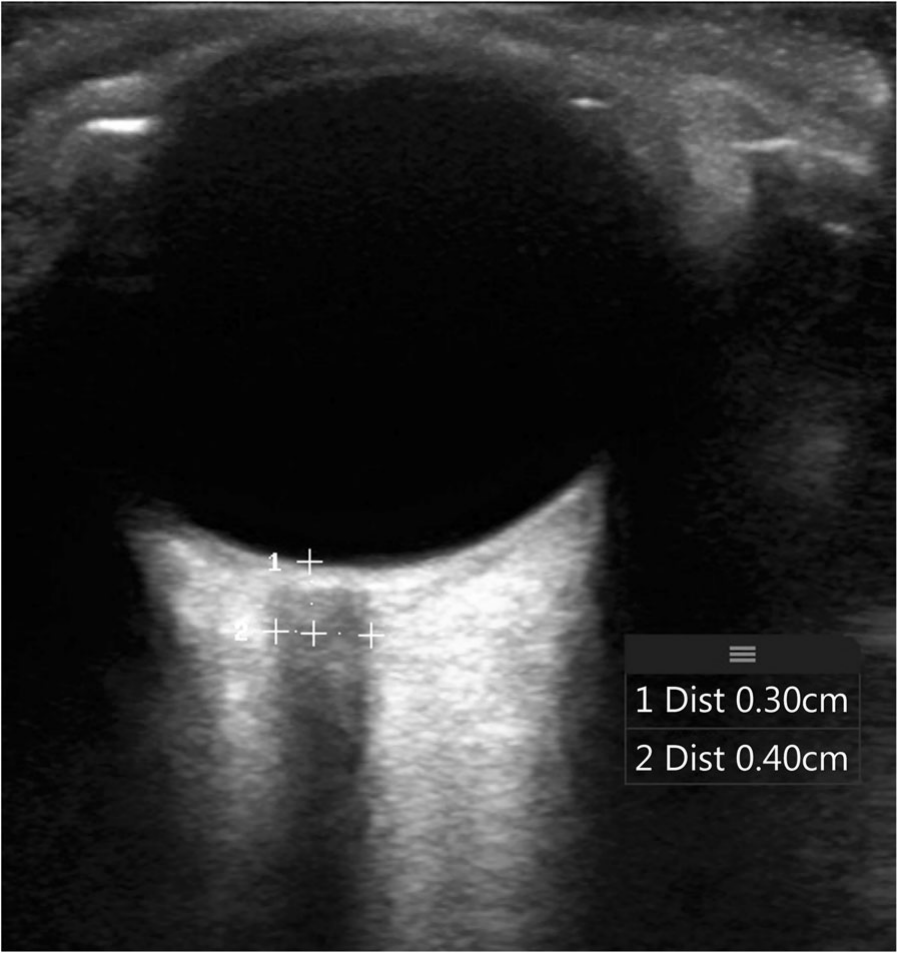
Optic nerve sheath diameter measurement. The optic nerve sheath is measured 0.3 cm posterior to the globe

Prior to performing the measurements, trainees were asked to rate their level of comfort with point-of-care ultrasound on a 5-point scale. They were also surveyed on the number of both general and ocular ultrasounds previously performed, in both the training and clinical setting. Trainees performed three optic sheath measurements of each eye with the volunteer trainee lying flat. All images were obtained in the axial plane, as the ultrasound probe was placed on the closed eyelid. An optimal image of the optic nerve was saved, and 3 mm posterior to the retina was measured (Fig. 1). The diameter of the optic nerve was then measured. Data were documented in deidentified form. These measurements were blinded from the instructors. The instructors then performed the same measurements on the same volunteer trainee immediately after. Data were analyzed with a 2 (eye: right vs. left) × 2 (experience level: trainee vs. expert) mixed design ANOVA, with repeated measures on the eye factor. A *P* value threshold of < 0.05 was interpreted as reaching statistical significance. Additionally, Bland-Altman plots were created to further explore the difference between the measurements taken by the experts vs. trainees, and the precision of the estimated limits of agreement was assessed by calculating their exact 95% confidence intervals [15, 16].

## Results

**Fig. 2:**
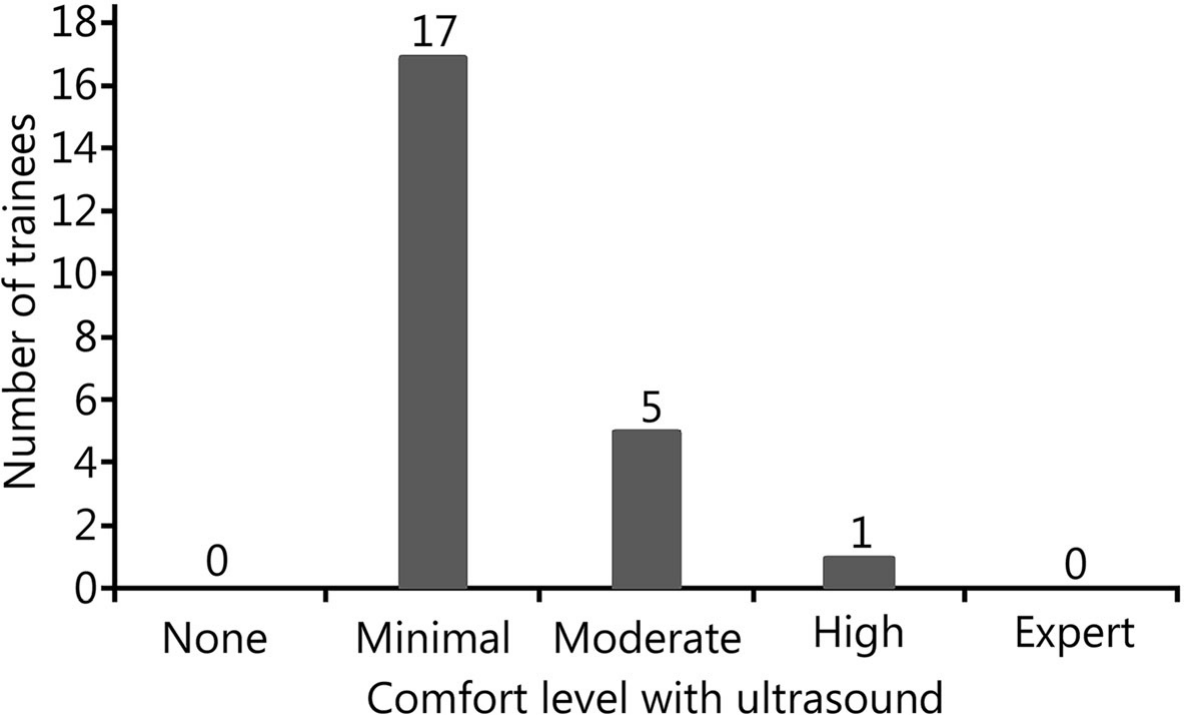
Comfort level of trainees with point-of-care ultrasound. The majority of trainees stated that their overall comfort level with point of care ultrasound was minimal, while a smaller percentage of trainees felt a moderate / high level of comfort

A total of 23 military trainees participated, along with four emergency physicians. Trainees had varying levels of comfort with performing point-of-care ultrasound (Fig. 2). The average comfort level on a scale of 1–5 was 2.25 among trainees. All trainees had minimal prior training in ultrasound, with each trainee having performed an average of 11.5 prior ultrasounds. The maximum ultrasound scans performed by any trainee was 20, while the minimum was 4. No trainee had performed more than one ocular ultrasound prior to this training session, and only one trainee had performed an ocular ultrasound.

Both the trainees and physicians obtained similar optic sheath measurements on the same volunteers, with the *M*_physician_ = 0.465 mm vs. *M*_trainees_ = 0.459 mm, *P* = 0.76. This shows a statistically nonsignificant difference of − 0.6 mm. Comparison of the contralateral eyes was not statistically significant between both the trainees and experts, *M*_Right_ = 0.463 vs. *M*_Left_ = 0.461, *P* = 0.72 (Table 1).

**Table 1:** Comparison between subjects with repeat measurements

Group	Eye	Mean ± SD	95% Confidence interval
Trainee	R	0.461 ± 0.014	0.433–0.490
	L	0.457 ± 0.012	0.432–0.482
Expert	R	0.465 ± 0.014	0.437–0.494
	L	0.465 ± 0.012	0.440–0.490

**Fig. 3:**
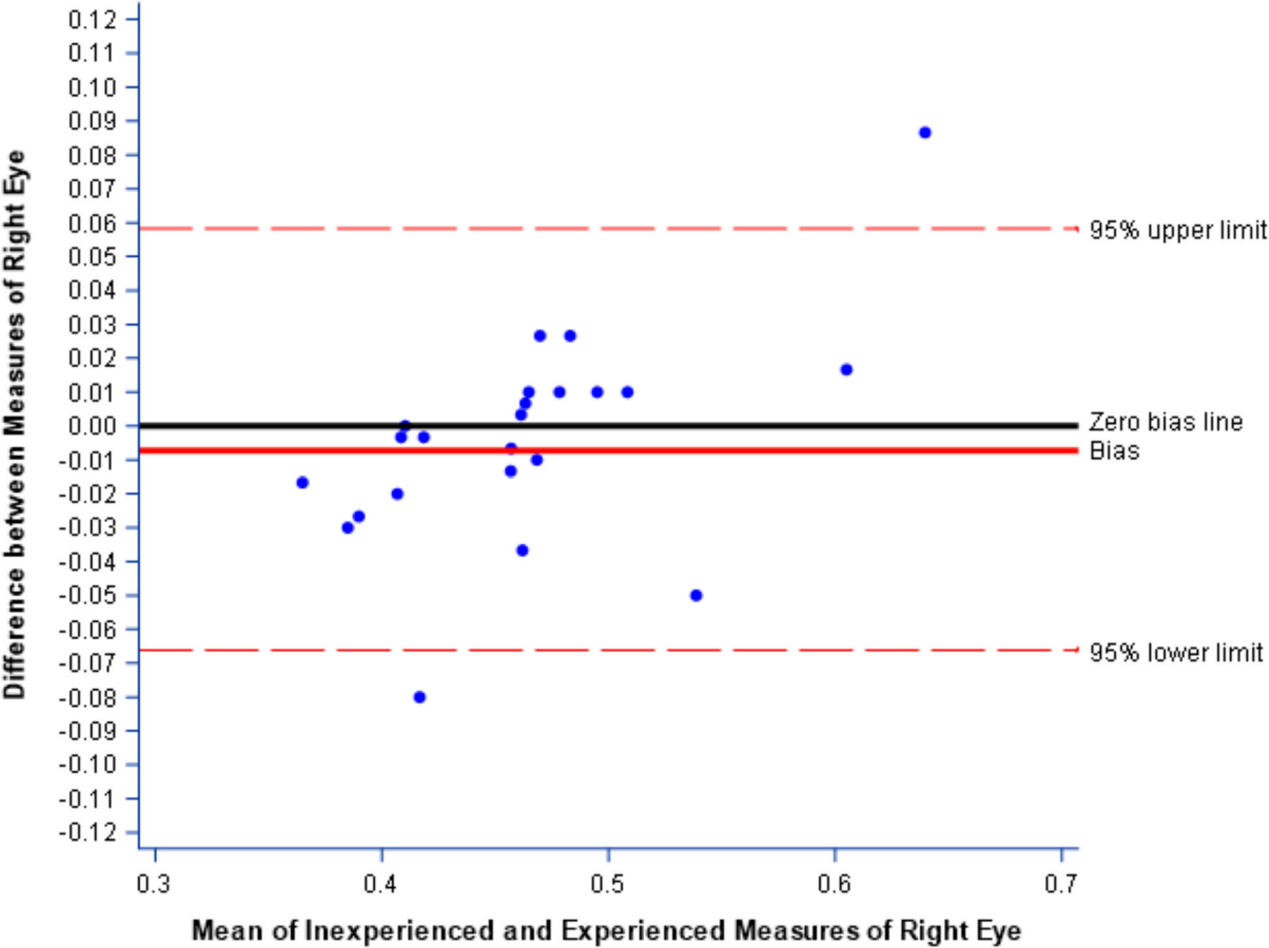
Bland-Altman plot for 95% limits of agreement for measures of right eye optic sheath: experienced and inexperienced users, indicating that measurements made by trainee users had minimal bias and were, on average, slightly lower than those from physician users

Using the Bland-Altman approach, the results showed that the mean difference between the measures of right eye optic sheath by trainee vs. physician users was − 0.004 mm (SE = 0.007, 95% CI = − 0.02 to 0.01, Fig. 3).

**Fig. 4:**
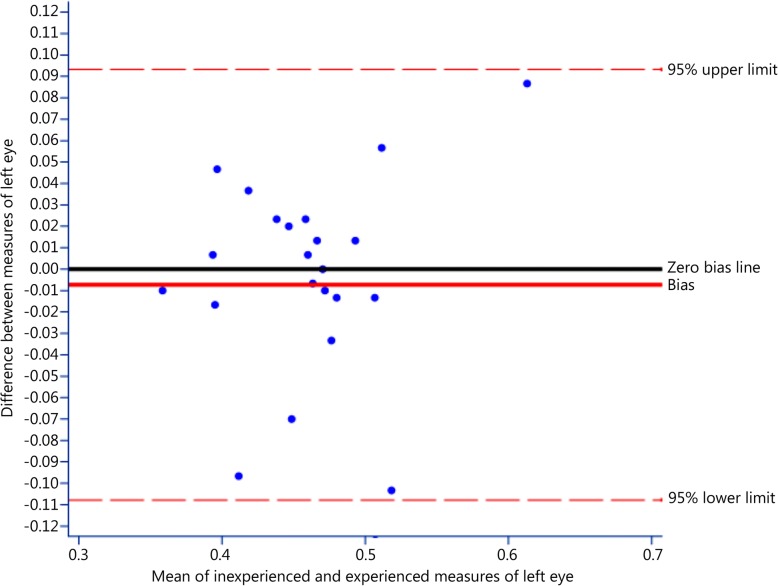
Bland-Altman plot for 95% limits of agreement for measures of left eye optic sheath: experienced and inexperienced users, indicating again that measures made by trainee users had minimal bias and were, on average, slightly lower than those from physician users

Also using the Bland-Altman approach, the results showed that the mean difference between the measures of left eye optic sheath by trainee vs. physician users was − 0.007 mm (SE = 0.01, 95% CI = − 0.03 to 0.01, Fig. 4).

To summarize these findings, 1) there were no statistically significant differences in measurements between military trainees and physicians; 2) measures of right and left eye optic sheath were, on average slightly lower for trainee vs. physician users; 3) the degree of bias in measures of optic sheath were slightly larger for the right compared to the left eye; and 4) the estimated lower and upper limits of agreement for measures of optic sheath were less precise for the left compared to the right eye. The main conclusion from our results is that the degree of bias in ONSD measures provided by trainees is very small.

## Discussion

In this study, we report the ability of inexperienced ultrasound users to perform ONSD measurements in live volunteers. SOCM trainees were able to perform the basic technique after a 5-min presentation and hands-on training session. Our brief survey demonstrates that the trainees were indeed inexperienced with point-of-care ultrasound, having performed fewer than 25 total scans previously. Performing twenty-five documented scans per specific modality is the current ACEP recommendation for emergency physicians [17]. Trainees additionally had no significant prior experience with ocular ultrasound specifically. Our results demonstrate no difference in ONSD measurements between trainees and experts. Additionally, there was no significant difference in measurements between contralateral eyes for either group.

Concerns related specifically to optic sheath ultrasound often include the narrow window for differentiation between normal and abnormal [18]. Our training specifically focused on slow, controlled movements while scanning for image optimization and on ensuring no visualization of the lens while measuring the ONSD. All images were obtained in the axial plane in this study. In prior studies, this is the most common approach. Some concerns have been raised that this approach is prone to error from shadowing by the lamina cribosa or refraction artifacts related to insonation through the lens. Prior literature has noted significant variation in cutoffs for normal/abnormal using this technique, and highly variable test characteristics of these different cutoff values [19–21]. ONSD can potentially be obtained in the coronal plane as well [22]. This technique may avoid some problems related to the axial approach. The coronal plane, while promising, is not yet well validated and was not performed in this study to maintain consistency.

Given the serious environment in which the military may be required to treat patients, portable ultrasound lends itself well to triage and diagnosis of multiple medical conditions when more advanced imaging is not available, or when repeat measurements may be of value in an ICU setting. There has been prior research evaluating the training regarding point-of-care ultrasound by military providers [23]. Prior military and combat training with point-of-care ultrasound regarding focused abdominal sonography in trauma (FAST), cardiac activity, pneumothorax, and fractures has been studied [24–26]. To our knowledge, this is the first study evaluating military providers’ ability to accurately measure ONSD. Previous literature has raised the question whether medics have the ability to accurately obtain ultrasonographic measurements compared to physicians [27]. We believe that our study helps to answer this question when applied to the ONSD modality.

Management of intracranial injuries is complex, and patients with such injuries often require care at a large tertiary care center with trauma and advanced radiographic capabilities. Our trainees may be required to treat patients who sustain severe intracranial injuries in remote areas, and high levels of resources for transport to a tertiary care center may be required. Multiple studies have been previously performed showing the practicality of point-of-care ultrasound for the military in austere locations [28–30]. Given the knowledge that trainees can perform this accurately and that point-of-care ultrasound has been shown to be useful in the combat environment, ocular ultrasound regarding ONSD may speed the time to diagnosis and delay the need for definitive transport.

Our study is not without limitations. The Mindray ultrasound machine used is not a device typically used in the military, and while portable, it is not a truly handheld device. Ultrasound scans were performed in a simulation center environment on healthy volunteers, which may limit external validity to clinical scenarios with potentially unstable patients. All volunteers were uninjured, with no current intracranial injuries. This additionally may remove some of the variability seen between injured and uninjured optic sheath measurements. Additionally, the instructors of the hands-on education session also served as the gold standard for measuring the diameter of the optic nerve sheath, which could lead to bias, although they were blinded from the trainees’ measurements.

## Conclusion

In summary, our study demonstrates that ONSD can be learned quickly by trainees with minimal prior experience in point-of-care ultrasound. Trainees were able to perform ONSD with an accuracy similar to ultrasound experts. This skill has the potential to diagnose severe intracranial injuries in low-resource environments areas and assist in rapid triage, treatment and evacuation decisions.

## Abbreviations

FAST: Focused abdominal sonography in trauma
ICP: Elevated intracranial pressure
ONSD: Optic nerve sheath diameter
SOCM: Special operations combat medic
